# Bacterial community profiles of select tributaries of Laguna Lake in the Philippines

**DOI:** 10.1128/mra.01161-23

**Published:** 2024-04-17

**Authors:** Arizaldo E. Castro, Andrew D. Montecillo, Ren Mark D. Villanueva, Marie Christine M. Obusan

**Affiliations:** 1 Microbial Ecology of Terrestrial and Aquatic Systems Laboratory, Institute of Biology, College of Science, University of the Philippines Diliman, Quezon City, Philippines; 2 Institute of Biological Sciences, College of Arts and Sciences, University of the Philippines Los Baños, Los Baños, Laguna, Philippines; Montana State University, Bozeman, Montana, USA

**Keywords:** Laguna Lake, bacterial community, emerging pollutants, microbial ecology, Philippines, metagenomic analysis

## Abstract

Laguna Lake is known for its ecological, economic, and cultural importance. Effects of urbanization and accumulation of emerging pollutants have been associated with its water quality; however, the microbial ecology of its tributaries remains to be explored. We report bacterial community profiles from shotgun metagenomes of its select tributary waters.

## ANNOUNCEMENT

Laguna Lake is a class C freshwater body that supports fisheries, agriculture, and livestock within nearby regions. It covers 911.7 km² and holds 3.2 billion cubic meters of water (1). It receives water from about 100 rivers and streams, mainly from the east. Urban rivers and waterways, serving as pollutant carriers from residential and commercial areas, affect the lake. Key tributaries of interest are those in Metro Manila and Laguna due to urbanization. Heavy metals and antibiotic-resistant bacteria (2), along with fecal indicators, have been found in the lake (3, 4). Therefore, analyzing its bacterial communities is crucial due to their role as pollution indicators. Three tributaries were profiled in this study: San Cristobal River (14.236944 N, 121.113611 E), Mangangate River (14.430278 N, 121.045 E), and Sapang Malapit Creek (14.571455 N 121.089539 E). San Cristobal River, one of the most polluted rivers influencing the lake’s health, contributes about 5% of Laguna Lake’s total water (5, 6). Mangangate River in Muntinlupa City, 10 km long, has high fecal contamination. Sapang Malapit Creek in Pasig City is a highly polluted waterway spanning 160 m in length (7). These tributaries collect waste from nearby agro-industrial and residential areas.

**Fig 1:**
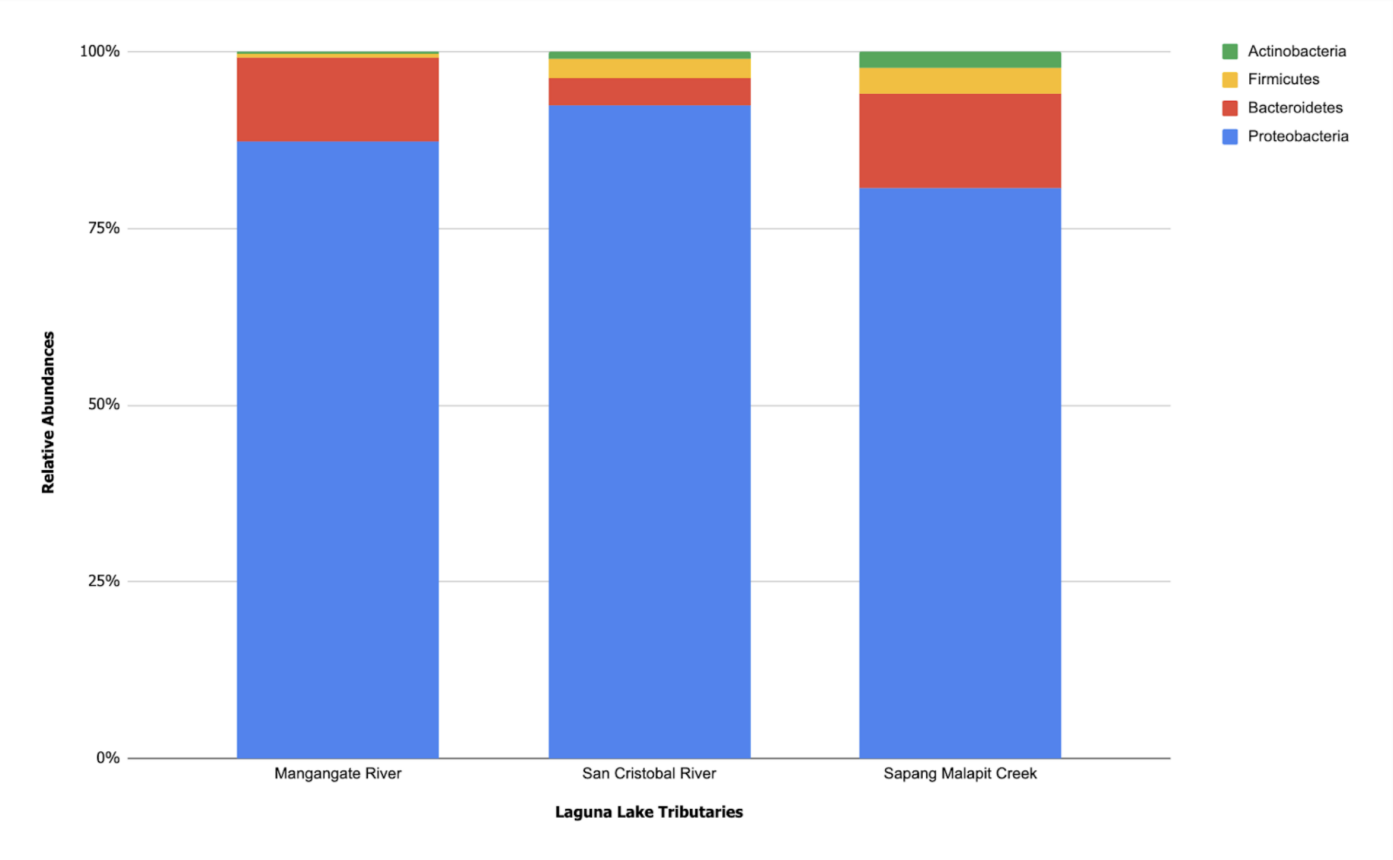
Phylum-level community profiles of Laguna Lake tributaries San Cristobal River, Mangangate River, and Sapang Malapit Creek.

Three samples per site were collected during the 2021 Philippine southwest monsoon. For each sample, 1,000 mL of water from 1 m depth was collected using sterile amber bottles. Samples were settled to remove suspended solids. After this, pre-filtration and vacuum filtration with 0.45 µm nitrocellulose membranes were performed, using 3,000 mL per site. Membrane filters were processed for total eDNA extraction using QIAGEN DNeasy PowerWater Kit immediately after filtration. Extracted DNA was stored at −20°C. Library preparation was performed using the Illumina Nextera XT DNA kit, and DNA sequencing was conducted on a NovaSeq 6000 platform (2 × 100 bp). Reads for all biological replicates per site were quality controlled with fastp (0.23.2), trimming adapters, and discarding low-quality sequences (<Q20). Reads were annotated using mOTUs2 (2.5.1) to determine taxonomic profiles using phylogenetic marker gene-based operational taxonomic units (mOTUs) at ≥97% minimum nucleotide identity. The metagenomes contained 98.46% to 99.56% bacteria and 0.09% to 0.37% archaea. Using the mean relative abundances of the three replicates per site, the major bacterial phyla included Proteobacteria (78.58% to 91.13%), Bacteroidetes (3.72% to 13.01%), Firmicutes (0.54% to 3.58%), and Actinobacteria (0.37% to 2.25%) (Fig. 1). At the species level, 918 mOTUs were identified. Across three sites, nine mOTUs had mean relative abundances over 2.00%: *Acinetobacter towneri* (2.00%), *Acinetobacter harbinensis* (2.09%), *Tolumonas auensis* (2.57%), *Bacteroides graminisolvens* (2.69%), *Parabacteroides chartae* (4.32%), *Sulfurospirillum* sp. (4.34%), *Acidovorax temperans* (5.51%), *Dechloromonas agitata* (11.56%), and *Aeromonas* sp. (12.21%).

The presence and abundance of potentially pathogenic bacteria, specifically *Acinetobacter* and *Aeromonas*, raise concerns about infectious microbes in Laguna Lake, considering its recreational and economic purposes.

## Data Availability

The raw sequences obtained in this project are available in the National Center for Biotechnology Information Sequence Read Archive (NCBI SRA) under the following accession numbers: SRR18609915 (San Cristobal River), SRR18609916 (Mangangate River), and SRR26638712 (Sapang Malapit Creek).
